# Infection status of human parvovirus B19, cytomegalovirus and herpes simplex Virus-1/2 in women with first-trimester spontaneous abortions in Chongqing, China

**DOI:** 10.1186/s12985-018-0988-5

**Published:** 2018-04-23

**Authors:** Ya-Ling Gao, Zhan Gao, Miao He, Pu Liao

**Affiliations:** 1 Clinical Medical School, Southwest Medical University, Luzhou, 646000 China; 2 The People’s Hospital of Chongqing, Chongqing, 400000 China; 3 Institute of Blood Transfusion, Chinese Academy of Medical Sciences, Chengdu, 610052 China; 4 The Sichuan Blood Safety and Blood Substitute International Science and Technology Cooperation Base, Chengdu, 610052 China

**Keywords:** Human parvovirus B19, Cytomegalovirus, Herpes simplex Virus-1/2, First-trimester spontaneous abortion, Real-time qPCR

## Abstract

**Background:**

Infection with Parvovirus B19 (B19V), Cytomegalovirus (CMV) and Herpes Simplex Virus-1/2 (HSV-1/2) may cause fetal loses including spontaneous abortion, intrauterine fetal death and non-immune hydrops fetalis. Few comprehensive studies have investigated first-trimester spontaneous abortions caused by virus infections in Chongqing, China. Our study intends to investigate the infection of B19V, CMV and HSV-1/2 in first-trimester spontaneous abortions and the corresponding immune response.

**Methods:**

100 abortion patients aged from 17 to 47 years were included in our study. The plasma samples (100) were analyzed qualitatively for specific IgG/IgM for B19V, CMV and HSV-1/2 (Virion\Serion, Germany) according to the manufacturer’s recommendations. B19V, CMV and HSV-1/2 DNA were quantification by Real-Time PCR.

**Results:**

No specimens were positive for B19V, CMV, and HSV-1/2 DNA. By serology, 30.0%, 95.0%, 92.0% of patients were positive for B19V, CMV and HSV-1/2 IgG respectively, while 2% and 1% for B19V and HSV-1/2 IgM.

**Conclusion:**

The low rate of virus DNA and a high proportion of CMV and HSV-1/2 IgG for most major of abortion patients in this study suggest that B19V, CMV and HSV-1/2 may not be the common factor leading to the spontaneous abortion of early pregnancy.

## Background

Spontaneous miscarriage, one of the most common pregnancy complications, is not only related to morbidity or mortality [1], but also has an obvious social and psychological impact on women [2]. The incidence of spontaneous miscarriages in pregnancies was reported to be as high as 15%, and at least 80% of those occurred in the first trimester of pregnancy [3]. There are many reasons leading to spontaneous abortions such as genetic factors, reproductive anatomical abnormalities, embryo factors and virus infections, in which virus infections have attracted more and more attention [4, 5]. Early spontaneous miscarriage (ESM) has many causes, such as uterine structural defects and chromosomal abnormalities. However, the cause of 40% of ESMs remains unclear [6]. Abnormal implantation, placentation or blood vessel transformation are thought to result in miscarriage [7, 8]. An active infection could interfere with the pregnancy by affecting any of the above-mentioned processes as well as disrupt the immune balance, whether it resulted in placental and fetal infection or not.

Some recent studies showed that viruses such as Human Parvovirus B19, cytomegalovirus (CMV), and herpes simplex virus (HSV-1/2) might be the pathogens of spontaneous abortion [9, 10]. Nonetheless, some studies suggested that B19V, CMV and HSV-1/2 infections were not commonly associated with first-trimester spontaneous [11–13]. Because of these, the relationship between B19V, CMV and HSV-1/2 and first-trimester spontaneous remains controversial.

It is well known that B19V belongs to Erythroviruses genus that is pathogenic for humans and may infects placenta [14–17]. Epidemiologic studies of B19V infection in China have been mostly reported in healthy blood donors, plasma pools [18–22] and in HIV-infected patients [23]. The prevalence of B19 DNA in HIV positive individuals is high with a positive rate of 4.5% [23], a higher prevalence than that in blood donors ranged from 0.06% to 3.51% [19, 20, 22] was reported. Relevant research shows that non-immune hydrops fetalis (NIHF) and intrauterine fetal death (IUFD) may be caused by fetal infection with B19V [24] and B19V infection-associated fetal death and hydrops fetalis occur mostly during the second trimester [25].

CMV is a ubiquitous virus with a seroprevalence 42.3–68.3% of pregnant women in developed countries [26, 27], but over 95% in developing countries such as China [28–30]. After primary infection, CMV can establish a lifelong latent infection that can be reactivated [31]. In addition to typical manifestation, such as nonspecific febrile disease or a mild self-limiting mononucleosis like syndrome, severe or prolonged symptomatic CMV infections are also reported [32, 33]. Primary CMV infection during pregnancy can cause congenital defects [34]. Seroepidemiological data are important for estimating the risk of primary CMV infection. However, available data for CMV infection during pregnancy in South China are inadequate. CMV seroprevalence in infants still needs to be clarified.

Besides, genital herpes has become an increasing common sexually transmitted infection in recent years. From the late 1970s, HSV-2 seroprevalence has increased by 30%, resulting that one out of five adults is infected [35, 36]. As for the pregnant population, there is a high prevalence of genital herpes. In Italy, the number of women who acquire HSV infection during pregnancy is about 3%. Among Italian pregnant women, the seroprevalence varies from 7.6% to 8.4% seroprevalence which is lower than that in US (22%). Guangdong, where is one of the few areas to report the epidemiological data in pregnant population, the prevalence of HSV-2 infection in pregnant women was 23.56% [37]. HSV-1/2 seroprevalence survey is limited to the special population in China, and the epidemic situation in the pregnant population is not clear [11, 38].

The relationship between first-trimester spontaneous abortions and B19V, CMV, and HSV-1/2 has not been investigated in Chongqing. Knowing the distribution of B19V, CMV, and HSV-1/2 in women with first-trimester spontaneous abortions in Chongqing City has great significance for preventing miscarriage and improving fetal survival rate. Therefore, we initiated this study to investigate the current infection status of B19V, CMV, and HSV-1/2 in women with first-trimester spontaneous abortions in order to catalyze the current policy resources required for that population in Chongqing, China.

## Methods

### Sample collection

Our samples came from two hospitals (Dianjiang county people’s hospital and the Chongqing Municipal People’s Hospital) in Chongqing. The whole blood samples (100) were collected from women, with a history of 0–4 spontaneous abortions, after the fetal loss. Whole blood samples were collected in one ethylenediaminetetraacetate-k2 (with separator gel) vacuum tubes (Greiner, Kremsmünster, Austria) at the blood collection sites. Plasma was separated from the RBCs by centrifuge. Samples were then frozen and shipped on dry ice to Institute of Blood Transfusion, Chinese Academy of Medical Sciences. Spontaneous abortions largely occurred from the fifth to eighth week of pregnancy, and not later than the twelfth week. The median age was 30.50 years, with a range of 17–47 years. Women younger than 30 years old accounting for a certain proportion (64%) in this study. All samples and detailed medical records were gathered at the Chongqing People’s Hospital from March 2013 to March 2015. The first-trimester was defined as less than 13 integral weeks in line with previous study [39].

### Viral DNA tests

The plasma samples were stored at − 20 °C after collected. DNA extraction was performed with a QIAamp DNA Blood Mini Kit (QIAGEN, Hilden, Germany). The isolated DNA was stored at − 80 °C before PCR analysis. Protocols for detection of B19V, CMV, and HSV-1/2 DNA in plasma were developed.

B19V, CMV and HSV-1/2 DNA were quantification by Real-Time PCR. For the detection of B19V, the forward primer S-B19-F and reverse primer S-B19-R were used to amplify a 133 bp fragment of the conserved region of the NS1 gene in the B19 genome [40]. The primers used for the detection of the CMV, termed SHCMV-F and S-HCMV-R, were used to amplify a 136 bp segment from the MIE gene [41]. HSV-1/2 DNA was detected using primers SP-HSV-F and SP-HSV-R, which amplified a 112 bp segment located in the most conserved region of the HSV DNA polymerase gene [42]. Negative controls that used water as template and positive controls that used 50 copies plasmid as template were also included in each run. The cycling conditions were as follows: 1 cycle of 95 °C for 10 min; 40 cycles of 95 °C for 15 s, 55 °C for 30 s, and 72 °C for 30 s; and a final cycle of 95 °C for 15 s, 60 °C for 15 s, with a gradual increase to 95 °C in 30 min at a ramp rate of 2% to get melting curves. The detailed primer sequences used are shown in Table 1. The sensitivity and stability of the qPCR detection system were also tested by serial dilution of each plasmid. The PCR product with restriction sites of the three viruses was ligated with pMD18-T easy Vector from Promega company to obtain the corresponding plasmid.

**Table 1:** Primers Used for Real-Time PCR

Primers	Sequences	Location	Amplicon length(bp)
S-B19V-F	5’-ACCAGTTCAGGAGAATCAT-3’	2256–2274	133
S-B19V-R	5’-CCCACACATAATCAACCC-3’	2371–2388	
S-HCMV-F	5’-GACTATCCCTCTGTCCTCAGTA-3’	171,231–171,252	136
S-HCMV-R	5’-AGACACTGGCTCAGACTTGA-3’	171,117–171,136	
SP-HSV-F	5’-CCGGAGAGGGACATCCAGGACTT-3’	65,876–65,898	112
SP-HSV-R	5’-GGGCCATGAGCTTGTAATACACCGT-3’	65,963–65,987	

### Serological tests

The plasma samples (100) were analyzed qualitatively for specific IgG/IgM for B19V, CMV and HSV-1/2 (Virion\Serion, Germany) according to the manufacturer’s recommendations. Samples were initially tested for one round, with a second test being conducted if the initial results were ambiguous. If the ambiguous result still remained, it was taken as the final result.

### Statistics analysis

All statistical analyses were conducted using SPSS 11.5 (SPSS, Inc., Chicago, IL). *P* value of less than 0.05 was considered to be statistically significant.

The study was approved by the ethics committee of the Chongqing People’s Hospital.

## Results

### Viral DNA study for B19V, CMV, and HSV-1/2

**Fig. 1:**
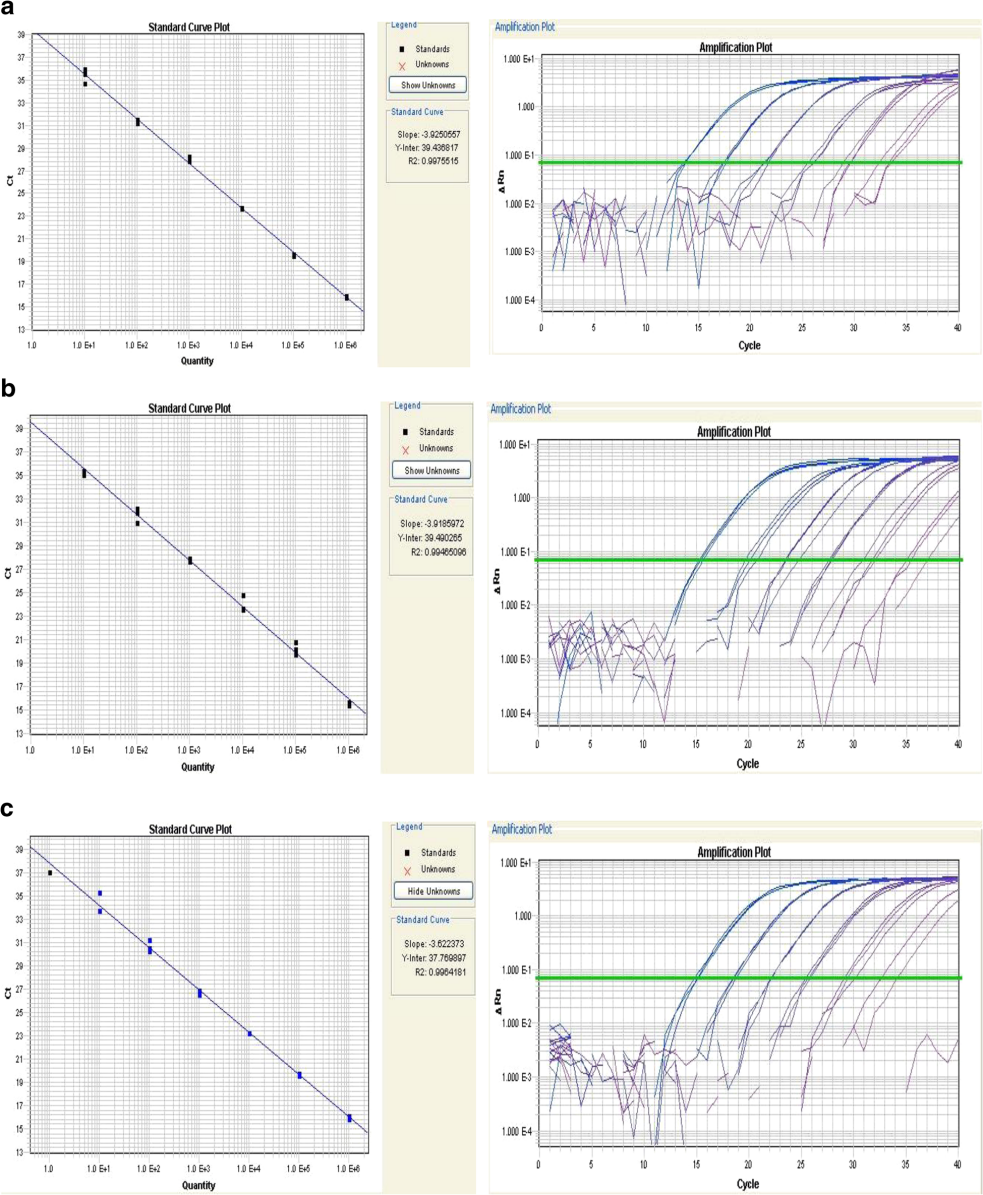
Determine the sensitivity of detection of B19V, CMV and HSV-1/2 by qPCR. **a**: A high coefficient of correlation (r2 = 0.9975) between the amplification cycle number (Ct values) and copy number representing the B19V virus titer. The standard curve indicates that qPCR can be used effectively to evaluate even low level of B19V DNA in patients. **b**: A high coefficient of correlation (r2 = 0.9946) between the amplification cycle number (Ct values) and copy number representing the CMV virus titer. The standard curve indicates that qPCR can be used effectively to evaluate even low level of CMV DNA in patients. **c**: A high coefficient of correlation (r2 = 0.9964) between the amplification cycle number (Ct values) and copy number representing the HSV-1/2 virus titer. The standard curve indicates that qPCR can be used effectively to evaluate even low level of HSV-1/2 DNA in patients

The serially diluted B19V, CMV and HSV-1/2 DNA standards of 1 × 101–106 copies per ul were added into the real-time PCR system 5ul each as templates to measure the intra and inter repeatability, detection limits and the reaction efficiency (Table 2 (a, b, c) and Fig. 1). The CT is cycle threshold and the CV is coefficient of variation. The largest CV is 4.80%, which means the differences within intra and inter were small. Thus, the DNA standard could successfully apply to quantitative PCR amplification system (Q-PCR) (Fig. 1). The detection limits of the real-time PCR were 50 copies per reaction volume for B19V, CMV and HSV-1/2. None of the specimens was positive for B19V, CMV, and HSV-1/2 DNA.

**Table 2:** The test of repeatability based on SYBR Green PCR system

a. The serially diluted B19V standards of 1 × 10^1^–10^6^ copies per ul to measure the intra and inter repeatability			
	Plasmid copies(cp/μl)	B19. Ct (‾x ± s)	B19. CV (%)
Inter Repeatability	1 × 10^6^	15.91 ± 0.07	0.46
	1 × 10^5^	19.60 ± 0.08	0.41
	1 × 10^4^	23.71 ± 0.05	0.21
	1 × 10^3^	28.12 ± 0.25	0.91
	1 × 10^2^	31.37 ± 0.20	0.64
	1 × 10^1^	35.45 ± 0.63	1.80
Intra Repeatability	1 × 10^6^	15.46 ± 0.66	4.80
	1 × 10^5^	19.86 ± 0.53	3.38
	1 × 10^4^	23.86 ± 0.23	1.04
	1 × 10^3^	27.99 ± 0.25	0.66
	1 × 10^2^	31.42 ± 0.17	0.41
	1 × 10^1^	35.21 ± 0.86	2.85
b. The serially diluted CMV standards of 1 × 10^1^–10^6^ copies per ul to measure the intra and inter repeatability			
	Plasmid copies(cp/μl)	CMV. Ct (‾x ± s)	CMV. CV (%)
Inter Repeatability	1 × 10^6^	15.52 ± 0.16	1.03
	1 × 10^5^	20.27 ± 0.5	2.50
	1 × 10^4^	24.02 ± 0.69	2.87
	1 × 10^3^	27.83 ± 0.15	0.52
	1 × 10^2^	31.65 ± 0.61	1.94
	1 × 10^1^	35.24 ± 0.23	0.64
Intra Repeatability	1 × 10^6^	15.80 ± 0.43	2.93
	1 × 10^5^	20.72 ± 0.71	3.10
	1 × 10^4^	24.43 ± 0.75	2.47
	1 × 10^3^	28.26 ± 0.60	2.18
	1 × 10^2^	32.36 ± 1.08	3.21
	1 × 10^1^	35.88 ± 0.79	2.23
c. The serially diluted HSV standards of 1 × 10^1^–10^6^ copies per ul to measure the intra and inter repeatability			
	Plasmid copies(cp/μl)	HSV. Ct (‾x ± s)	HSV. CV (%)
Inter Repeatability	1 × 10^6^	16.03 ± 0.16	1.01
	1 × 10^5^	19.67 ± 0.11	0.56
	1 × 10^4^	23.25 ± 0.03	0.11
	1 × 10^3^	26.73 ± 0.18	0.66
	1 × 10^2^	30.69 ± 0.52	1.70
	1 × 10^1^	34.52 ± 1.06	3.07
Intra Repeatability	1 × 10^6^	16.73 ± 0.68	4.32
	1 × 10^5^	20.31 ± 0.63	3.20
	1 × 10^4^	24.02 ± 0.74	3.26
	1 × 10^3^	27.51 ± 0.75	2.90
	1 × 10^2^	31.48 ± 0.85	2.54
	1 × 10^1^	35.84 ± 1.22	3.17

*Ct* Cycle threshold, *CV* Coefficient of Variation

### Serologic study for B19V, CMV, and HSV-1/2

In the serologic study, CMV IgG had the highest rate of positivity (95.0%), followed by HSV-1/2 IgG (92.0%) and B19V IgG (30.0%) (Table 3). Specific IgG antibodies against CMV and HSV-1/2 were present at a high rate in the population studied, which indicated that a large proportion of the participants had been infected by CMV and HSV-1/2 in the past. Women aged from 23 to 40 were more likely to experience past B19V infections than women in other ages. In addition, 2% and 1% first-trimester spontaneous abortion samples were positive for B19V and HSV-1/2 IgM respectively (Table 3), which might indicate that the women were undergoing a recent B19V and HSV-1/2 infections. CMV IgM, which is used to screen acute CMV infections for hospital patients, is negative in this study.

**Table 3:** Serologic Study for B19V, CMV and HSV1/2 in Women With First-Trimester Spontaneous Abortions

Maternal age	Median	Number of cases (%)	B19 IgG (%)	HSV IgG (%)	CMV IgG (%)	B19 IgM (%)	HSV IgM (%)
< 30	23.00	67 (67.00)	19 (19.00)	66 (66.00)	67 (67.00)	2 (2.00)	0 (0)
30–40	35.00	25 (25.00)	9 (9.00)	20 (20.00)	23 (23.00)	0 (0)	1 (1.00)
> 40	43.00	8 (8.00)	2 (2.00)	6 (6.00)	5 (5.00)	0 (0)	0 (0)
total	30.50	100 (100.00)	30 (30.00)	92 (92.00)	95 (95.00)	2 (2.00)	1 (1.00)

## Discussion

Spontaneous abortion is a common problem in early pregnancy. Spontaneous miscarriages occur in approximately 14% to 16% of naturally conceived pregnancies [43] and approximately 15% of clinically recognized first-trimester pregnancies undergo miscarriage [44]. Loss of subclinical pregnancy is even higher, and is reported to be approximately 60% based on the measurement of human chorionic gonadotrophin levels [45]. Abortions may arise from an abnormal uterine cavity, parental karyotypes, endocrine factors, infection, and autoimmunity [46, 47]. Although causal relationships between abortion and infections are difficult to establish, the detection rate of B19V, CMV, and HSV during pregnancy is an important way to analyze their relationship with first-trimester spontaneous abortion.

In this study, in a total number of 100 cases, none of the specimens were positive for B19V, CMV, and HSV-1/2 DNA. These negative results for virus DNA may indicate that first-trimester spontaneous abortions associated with B19V, CMV, or HSV infection are not common in Chongqing, which is somewhat consistent with several previous studies [11–13, 39]. Our findings supported results by another study in nearby regions, which after measuring B19V, CMV, and HSV-1/2 DNA in 1716 plasma specimens, found that none of the specimens were positive for B19V or CMV DNA and seven out of the 1716 specimens were positive for HSV DNA [11]. One study in Sweden measured B19V DNA in placental tissue also found a low frequency of B19V infection in first-trimester fetal loss (3%) [39]. Other studies investigating the presence of CMV DNA using PCR in human aborted material also failed to support a role for CMV infection in first-trimester spontaneous abortions [12, 13]. However, this finding is different from other surveys which reported that B19V, CMV, and HSV-1/2 infections are associated with the increased risk of first-trimester spontaneous abortions [48–50]. For example, Lassen et al. examined 2918 women, and found a correlation between acute B19V infection during the first trimester of pregnancy [49]. Besides, Kapranos et al. reported a significant role of HSV in first-trimester spontaneous abortion, which indicated that 41 of 95 cases (43.2%) of early spontaneous abortion showed signs of HSV infection compared with 36 of 216 (16.7%) cases of elective pregnancy termination [51]. The type of specimen, such as plasma, fetal tissues, bone marrow, or placenta, may be one of the main reasons for this diversity of these association between B19V, CMV, and HSV and first-trimester spontaneous abortions. Although the number of patients who experience first-trimester spontaneous abortions are insufficient in this study, we still believe that the relationship between infections and abortions are at least not directly related to Chongqing. Thus, a larger scale multi-center investigation should be performed in our further research to clarify this association.

Maternal sera were simultaneously examined for the detection of specific IgG and IgM antibodies against B19V, HSV-1/2, and CMV, which indicate past and acute infection respectively. Although no specimens were positive for B19V, CMV, and HSV-1/2 DNA, 30.0%, 95.0%, 92.0% of patients were positive for B19V, CMV and HSV-1/2 IgG respectively. Besides, 2% and 1% for B19V and HSV-1/2 IgM, which is lower than the reported healthy population [22, 52, 53]. This situation seems to be contradictory but can be explained. The positive B19V, CMV, and HSV IgG could be explained by the fact that primary infection is usually acquired during childhood, which means that the risk of primary infection is lower during pregnancy. The low proportion of IgM could be attributed to the low viral load of B19V, CMV, and HSV in acute infection which could be easily cleared by the autoimmune system in pregnant women. In addition, for B19V, one study found that an underlying cause of persistence might be quantitative or qualitative deficits in humoral response to B19V [54]. Because some persistently infected individuals have no production of antibodies to B19V (a quantitative defect), whereas others produce B19V IgM antibodies that could not neutralize the virus (a qualitative defect), which might be the reason of relatively low proportion of B19V IgG and IgM.

## Conclusions

Viral DNA or specific IgG and IgM antibodies detected in this study cannot indicate a potential pathogenic association between B19V, CMV, or HSV infection and spontaneous abortion in the first trimester in women from Chongqing. Further large case-control studies are required to elucidate the possible relationship between viral infections and pregnancy outcomes in Chongqing city. And positive plasma may not always represent fetal infection because of placental barrier, thus fetal tissues or placental should be required to clarify whether certain infections do increase miscarriage risk. We believe this analysis will catalyze the current policy resources required for spontaneous abortion prevention in Chongqing, China.

## Abbreviations

B19V: Parvovirus B19
CMV: Cytomegalovirus
ESM: Early spontaneous miscarriage
HSV-1/2: Herpes Simplex Virus-1/2
IgG: Immunoglobulin G
IgM: Immunoglobulin M
IUFD: Intrauterine fetal death
NIHF: Non-immune hydrops fetalis
real-time qPCR: Real-time Quantitative PCR Detection System
